# High Temporal Resolution Dual-Source Photon-Counting CT for Coronary Artery Disease: Initial Multicenter Clinical Experience

**DOI:** 10.3390/jcm11206003

**Published:** 2022-10-11

**Authors:** Martin Soschynski, Florian Hagen, Stefan Baumann, Muhammad Taha Hagar, Jakob Weiss, Tobias Krauss, Christopher L. Schlett, Constantin von zur Mühlen, Fabian Bamberg, Konstantin Nikolaou, Simon Greulich, Matthias F. Froelich, Philipp Riffel, Daniel Overhoff, Theano Papavassiliu, Stefan O. Schoenberg, Sebastian Faby, Stefan Ulzheimer, Isabelle Ayx, Patrick Krumm

**Affiliations:** 1 Department of Diagnostic and Interventional Radiology, Medical Center-University of Freiburg, Faculty of Medicine, University of Freiburg, Hugstetter Str. 55, 79106 Freiburg, Germany; 2 Department of Diagnostic and Interventional Radiology, University of Tuebingen, Hoppe-Seyler-Str. 3, 72076 Tuebingen, Germany; 3 First Department of Medicine-Cardiology, University Medical Centre Mannheim, and DZHK (German Centre for Cardiovascular Research), Partner Site Heidelberg/Mannheim, Theodor-Kutzer-Ufer 1-3, 68167 Mannheim, Germany; 4 Department of Cardiology and Angiology I, Medical Center-University of Freiburg, Faculty of Medicine, University of Freiburg, Hugstetter Str. 55, 79106 Freiburg, Germany; 5 Department of Cardiology and Angiology, University of Tuebingen, Otfried-Müller-Str. 10, 72076 Tuebingen, Germany; 6 Department of Radiology and Nuclear Medicine, University Medical Center Mannheim, Heidelberg University, Theodor-Kutzer-Ufer 1-3, 68167 Mannheim, Germany; 7 Computed Tomography, Siemens Healthcare GmbH, 91301 Forchheim, Germany

**Keywords:** photon counting, CT, coronary, cardiac, spectral imaging

## Abstract

The aim of this paper is to evaluate the diagnostic image quality of spectral dual-source photon-counting detector coronary computed tomography angiography (PCD-CCTA) for coronary artery disease in a multicenter study. The image quality (IQ), assessability, contrast-to-noise ratio (CNR), Agatston score, and radiation exposure were measured. Stenoses were quantified and compared with invasive coronary angiography, if available. A total of 92 subjects (65% male, age 58 ± 14 years) were analyzed. The prevalence of significant coronary artery disease (CAD) (stenosis ≥ 50%) was 17% of all patients, the range of the Agatston score was 0–2965 (interquartile range (IQR) 0–135). The IQ was very good (one, IQR one–two), the CNR was very high (20 ± 10), and 5% of the segments were rated non-diagnostic. The IQ and assessability were higher in proximal coronary segments (*p* < 0.001). Agatston scores up to 600 did not significantly affect the assessability of the coronary segments (*p* = 0.3). Heart rate influenced assessability only at a high-pitch mode (*p* = 0.009). For the invasive coronary angiography (ICA) subgroup (*n* = nine), the diagnostic performance for CAD per segment was high (sensitivity 92%, specificity 96%), although the limited number of patients who underwent both diagnostic modalities limits the generalization of this finding at this stage. PCD-CCTA provides good image quality for low and moderate levels of coronary calcifications.

## 1. Introduction

Diagnostic imaging by coronary computed tomography angiography (CCTA) has been established clinically with class one indication in international guidelines for the detection and exclusion of significant coronary artery disease (CAD) in patients with low and intermediate pretest probabilities [1,2]. However, one main limitation of CCTA imaging remains, namely the reduced ability to quantify calcified plaque stenosis, which often leads to an overestimation [3]. This may be attributed to the partial volume averaging of different densities within a single voxel and the beam-hardening artifacts of high-density calcifications, especially in combination with motion artifacts, which results in an overestimation of the calcified lesion’s size [4]. Therefore, photon-counting detector (PCD) CT is a promising method to improve image quality in CCTA [5]. Compared with energy-integrating detectors, which use an indirect conversion technology to detect the incident photons, PCD technology uses semiconductors that directly convert X-ray photons into electrical signals [6]. The advantages of PCD CT are reduced electronic noise, an increased contrast-to-noise ratio, improved spatial resolution, and the availability of spectral information in all standard acquisitions [7,8]. Previous PCD CT prototypes included cardiac gating in principle, but coronary diagnostics have been challenging because the temporal resolution was limited by a low gantry rotation speed [8]. Recently a new dual-source PCD CT prototype (NAEOTOM Alpha, Siemens Healthineers, Forchheim, Germany) has been cleared for clinical use which combines the temporal resolution of third-generation dual-source scanners with two PCD arrays providing all of the capabilities of PCD imaging [7,9,10]. CCTA may be acquired with the high spatial resolution of the PCDs while enabling the highest technically available temporal resolution.

The aim of our multicenter study is to systematically analyze the feasibility of PCD-CCTA at this scanner regarding the diagnostic image quality of coronary segments, vessels, and total CCTA scans on a larger study size in a clinical setting.

## 2. Material and Methods

### 2.1. Study Population

We retrospectively enrolled symptomatic subjects with chronic coronary syndrome who were clinically referred for cardiac CT angiography in the settings of first presentation and known coronary artery disease between January and March 2022. Subjects were excluded if they had contraindications to iodinated contrast administration and/or CT imaging in general. The study was approved by the ethics committee and all participants provided written informed consent.

### 2.2. CT Imaging

All patients underwent spectral photon-counting electrocardiogram (ECG)-synchronised CCTA (NAEOTOM Alpha, Siemens Healthineers, 2 × 120 kVp, collimation: 2 × 144 × 0.4 mm, z-coverage: 144 × 0.4 mm = 57.6 mm, pitch: 3.2, maximum table speed 737 mm/s, gantry rotation time 0.25 s, temporal resolution 66 ms, matrix 512 × 512, software version VA40) at the radiology departments of the three university hospitals: Tuebingen (*n* = 29), Freiburg (*n* = 29), and Mannheim (*n* = 34).

In the absence of contraindications, patients were prepared with sublingual nitrates (5 mg, isosorbide dinitrate) and intravenous beta blockers (5–15 mg, metoprolol) if necessary for diagnostic image quality, in line with current guidelines [11]. Their heart rates (HR) were determined at the time of scanning after premedication with beta blockers. HR variability (HV) was calculated as HV = (HRmax − HRmin)/[(HRmax + HRmin)/2].

Calcium scoring was performed as a non-contrast enhanced high-pitch scan with 120 kV. All patients received CCTA regardless of their Agatston score. The CCTA scan mode was dependent on HR with: stable HR < 65/min: high-pitch mode; HR 65/min–70/min: sequential mode with diastolic acquisition window (60–80% of RR-interval); HR > 70/min or high-HR variation (>10/min): sequential mode with extended systolic and diastolic acquisition window (30–80% of RR-interval); tachyarrhythmia > 90/min: low-pitch retrospective gating. Automatic tube current modulation (CARE Dose4D, Siemens Healthineers) was enabled, and the tube voltage was set to 120 kV for spectral imaging.

CCTA scans were initiated by bolus tracking or by the test bolus technique. Iodine contrast agent (Ultravist 370, Bayer Schering Pharma, Berlin, Germany; iopromide 769 mg/mL or iomeprol 400 mg J/mL, Bracco Imaging Deutschland GmbH, Konstanz, Germany) was administered depending on the patient’s body weight using an automated power injector (60–80 mL, 5–6 mL/s), followed by a saline flush (60–80 mL, 5–6 mL/s).

CCTAs were reconstructed with a slice thickness and increment of 0.4/0.3 mm, monoenergetic energy level 60 keV, quantum iterative reconstruction (QIR) level 3, vascular convolution kernel (Bv40), image matrix of 512 × 512, and a field-of-view restricted to the heart.

The effective tube current (mAs), CTDI_vol_ (mGy) and DLP (mGy.cm) were derived from the patient protocols. The effective dose (mSv) was calculated by the multiplication of the DLP with 0.015 mSv/(mGy.cm).

### 2.3. Image Evaluation

Two experienced radiologists (3–12 years of experience in CCTA imaging) at each center, who were blinded to the patient’s histories, prior imaging examinations, and clinical symptoms, analyzed PCD-CCTA data sets on a dedicated workstation (syngo.via, Siemens Healthineers) in consensus.

### 2.4. Definition of Evaluated Parameters

The image quality (IQ) of every coronary segment was rated using a five-point Likert scale. A score of 1 (*excellent*) was defined as the absence of motion artifacts and very good opacification of the coronary arteries; a score of 2 (*good*) as minor-movement artifacts or slightly reduced enhancement; a score of 3 (*fair*) for moderate-movement artifacts or reduction of contrast; a score of 4 (*poor*) indicated pronounced-movement artifacts such as doubling of the coronary arteries or low contrast, which might reduce reader certainty but was still diagnostic by consensus; a score of 5 (*non-diagnostic*) was given for an unreadable dataset due to movement or low contrast. Reduced assessability due to calcified plaques was not included in the IQ but was assessed separately, as described below.

Non-diagnostic coronary segments due to movement or low contrast corresponded to IQ = 5.

The assessability of coronary segments (diagnostic/non-diagnostic) due to coronary artery calcifications (CAC) was examined independently of the IQ rating. Segments were rated to be “non-diagnostic due to CAC” if at least one of the readers could neither exclude nor confirm ≥ 50% diameter stenosis due to calcified plaques [12].

The total assessability of the coronary segments (diagnostic/non-diagnostic) was derived from both of the aforementioned attributes considering any reasons rendering segments non-diagnostic.

The non-assessability of a coronary vessel was assumed if ≥1 segment in a main vessel (left anterior descending artery (LAD), left circumflex artery (LCX), or right coronary artery (RCA)) was non-diagnostic.

The non-assessability of whole PCD-CCTA scans for any reason was assumed if ≥1 segment of the complete coronary tree was non-diagnostic.

The contrast-to-noise ratio (CNR) was determined for each of the three main coronary vessels (LAD, RCA, LCX) and calculated by CNR = (average luminal HU − average perivascular epicardial HU)/(aortic SD HU), as described previously [13]. Luminal HU measurements were performed in all middle segments of the three main coronary arteries using a circular region of interest (ROI), including as much vessel lumen as possible without including the vessel wall or coronary plaques. A perivascular reference measurement was performed in the corresponding epicardial fat tissue next to the vessel measurement using an ROI of the same size, excluding the vessel wall or coronary plaques.

The degree of coronary artery luminal stenosis was measured in CCTA perpendicular to the vessel centerline on curved multiplanar reconstructions and in invasive coronary angiography (ICA). Significant CAD was defined as luminal diameter stenosis ≥50%. Non-diagnostic segments at CCTA were rated as potential significant CAD.

### 2.5. Statistical Analysis

Normally distributed continuous variables were expressed as mean (standard deviation) and were compared with Student’s *t*-test. Ordinal variables were expressed as median (interquartile range) and compared with Wilcoxon’s rank-sum test/Wilcoxon’s signed-rank test for independent/dependent samples. Categorical variables were summarized as absolute and relative frequencies and compared with the Chi-square test or Fisher’s exact test (expected frequency < 5). IQ, CNR, Agatston score, and the assessability of the vessels and segments were compared with Friedman’s test and Cochrane’s Q test, and the frequencies of diagnostic coronary segments (among Agatston and HR categories) with Kruskal–Wallis test. Cutoffs (Agatston score and HR) for the comparison of diagnostic assessability per patient were derived from the per-segment analysis. The correlation of the effective radiation dose with HR and HV was performed with Spearman’s rank correlation test. To assess the association between image quality and clinical parameters including gender, age, body mass index (BMI), HR, HV, and Agatston score, we fitted linear regression models, including the image quality as the outcome of interest and patient characteristics as covariates. Diagnostic performance was measured by sensitivity, specificity, accuracy, and positive- and negative-predictive values. Statistical tests were one- or two-sided, as appropriate. A *p*-value ≤ 0.05 was considered significant. All analyses were performed with statistical software SPSS Version 28.0.1.0 (IBM, Armonk, NY, USA).

## 3. Results

### 3.1. Baseline Characteristics

PCD-CCTA was successfully acquired in 92 subjects (65% male, mean age: 58 ± 13.7), comprising 1288 analyzed coronary segments. The Agatston scores had a wide range of 0–2965 (IQR 0–135), 8.9% of patients had known CAD, and 4% had coronary stents. All baseline characteristics are summarized in Table 1.

### 3.2. Patient Outcome

The overall prevalence of significant CAD was moderate (16 patients, 17%). Nine patients (10%) underwent invasive coronary angiography (ICA), which resulted in percutaneous coronary intervention in three patients (3%). The other patients received functional imaging to exclude the hemodynamic relevance of CAD.

### 3.3. Radiation Dose

The median DLP was 90.9 mGy.cm (IQR 52.8–235.5 mGy.cm), corresponding to a median effective radiation dose of 1.4 mSv (IQR 0.8–3.5 mSv) for spectral PCD-CCTA, which is below the typical dose for CCTA as reported by the PROTECTION VI registry (median DLP 195 mGy.cm, range of 3–5 mSv) [14]. Most examinations (60%) were below 2 mSv. A total of 49 patients were scanned in high-pitch mode, 36 in sequential mode, and 7 in retrospective mode. The radiation dose depended strongly on the scan mode with a mean effective dose of 1.0 ± 0.8 mSv for the high-pitch, 4.8 ± 4.0 mSv for the sequential, and 9.6 ± 4.4 mSv for the low-pitch retrospective mode (*p* < 0.001 for each paired comparison). The scan modes and dose modulation were chosen according to the patient’s HR and HV. Consequently, the radiation dose correlated with HR (*p* = 0.30; *p* = 0.004) and HV (*p* = 0.41; *p* < 0.001).

### 3.4. Image Quality and Assessability of Coronary Segments

**Figure 1:**
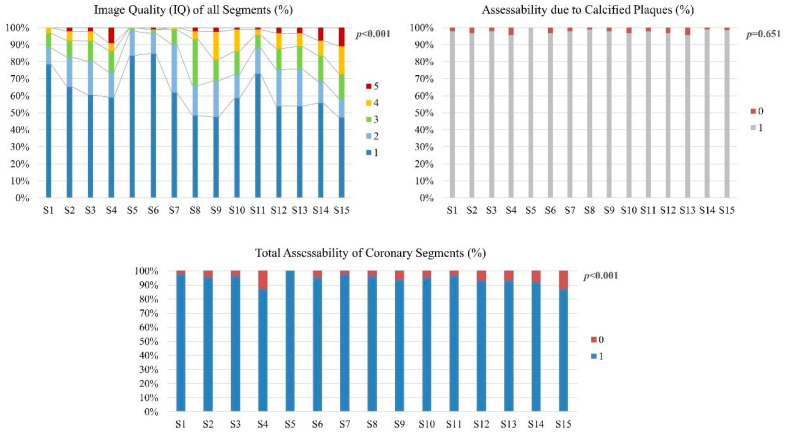
Image quality (IQ) of all coronary segments, assessability of segments due to calcified plaques, and total assessability of all coronary segments are shown. IQ and total assessability (considering image degradation due to movement, contrast, and calcifications) were significantly lower for distal segments. Assessability of calcified plaques did not differ among segments.

The overall IQs of the segments were very good (median one, IQR one–two), with an overall 5.1% of all coronary segments rated non-diagnostic due to any reason. A total of 2.9% of the segments were non-diagnostic due to movement or contrast, and 2.4% of segments were non-diagnostic due to calcified plaques. The IQ was lower in the distal (4/8/9/14/15) than in the proximal segments (1/5/6/7/11) (*p* < 0.001), corresponding to a significantly lower total assessability in distal segments (*p* < 0.001), which was not related to coronary calcifications (*p* = 0.7) but to movement or low contrast (*p* < 0.001) (Figure 1, Table 2).

**Figure 2:**
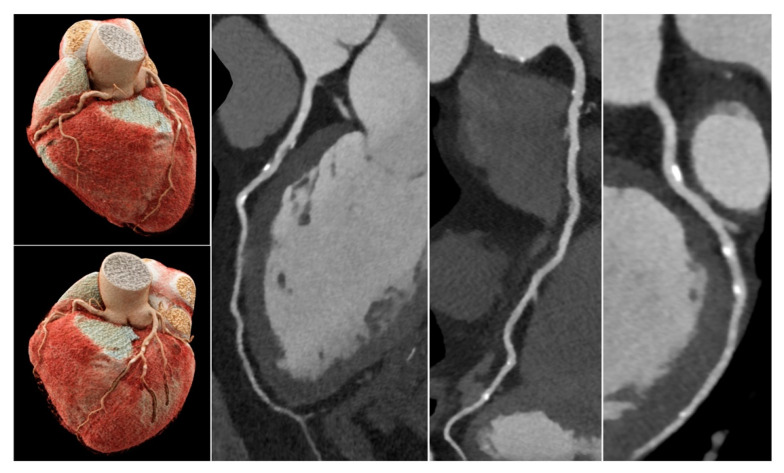
CCTA scan of a 75-year-old male patient with atypical angina pectoris. Risk factors: smoking and hypertension. Normal LV-function, normal resting electrocardiogram (ECG). Unspecific T-wave changes on exercise ECG. Total Agatston score was 589, which corresponded to the 68. percentile (multi-ethnic study of atherosclerosis) [15]. Sequential scan with diastolic acquisition window was performed at heart rate 68/min. All segments were diagnostic, IQ was 1–2. Despite a high plaque burden, all calcified plaques were assessable and could be rated as <50% diameter stenosis, so that significant CAD could be excluded with high certainty.

**Figure 3:**
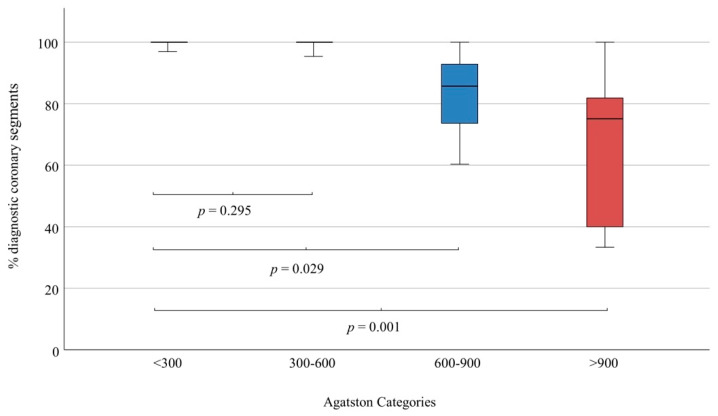
Frequency of diagnostic coronary segments depending on Agatston score. At Agatston scores < 600, assessability of segments remained very high (>95%) and did not differ between low (<300) and moderate (300–600) Agatston scores. At Agatston scores > 600, assessability of segments began to decline significantly, which aggravated at very high Agatston scores (>900).

The frequency of non-diagnostic coronary segments per patient was independent of small or moderate amounts of calcifications (Figure 2 and Figure 3) up to an Agatston score of 600 (*p* = 0.30), but increased significantly with a high Agatston score > 600 (*p* = 0.03) and very high Agatston score > 900 (*p* = 0.001).

**Figure 4:**
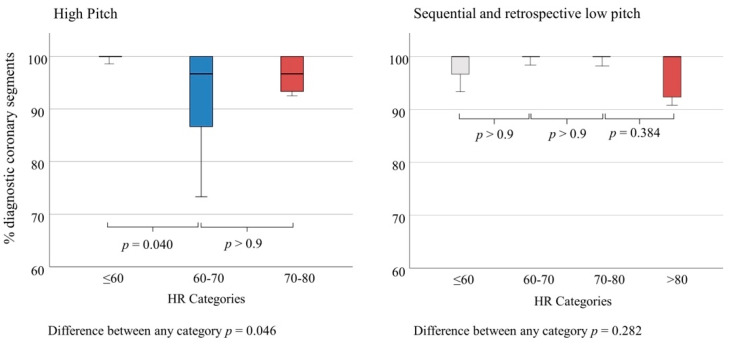
Frequency of diagnostic coronary segments depending on heart rate (HR) for high-pitch mode versus sequential and retrospective low-pitch modes. At high-pitch mode, assessability of segments began to decline significantly for HR > 60. Only a few patients at HR 70–80/min were scanned at high-pitch mode, hence further decline could not be shown on a significant level. At sequential and low-pitch retrospective modes, the frequency of diagnostic segments remained very high at HR up to 80/min. At HR > 80/min, a tendency towards lower assessability of segments could be observed, but this could not be proven on a significant level due to few examinations with very high heart rates.

A HR > 60/min resulted in more non-diagnostic segments for high-pitch mode (*p* = 0.04), but not for sequential and low-pitch modes (*p* > 0.9) (Figure 4).

### 3.5. Image Quality, Assessability and CNR of the Three Main Vessels

Total assessability was not significantly different among the three main vessels (87%, 87%, 86%, *p* = 0.9). That also applied separately for the assessability due to movement or contrast (*p* = 0.5) and the assessability due to coronary calcifications (*p* = 0.4), although the Agatston score was higher in the LAD than in the RCA (*p* = 0.001) and the LCX (*p* < 0.001) (Table 3).

### 3.6. Assessability of Whole Spectral PCD-CCTA Scans

Non-diagnostic coronary segments (≥1 segment) were present in 23 patients. Thereof 11 patients had significant CAD in other segments, such that CAD could still be confirmed on the patient level. In the remaining 12 patients (13%), CAD could neither be confirmed nor excluded by CCTA.

Comparing clinical parameters between patients with and without totally diagnostic CCTA scans, the HR (*p* = 0.044) and Agatston score (*p* = 0.001) were different for scans in high-pitch mode, whereas only BMI (*p* = 0.013) and Agatston score (*p* = 0.043) were different for scans in sequential and low-pitch modes (Table 4).

In multiple linear regression analysis, HR and HV were the only significant predictors of IQ in high-pitch mode but not in sequential mode (Supplementary Table S1).

**Figure 5:**
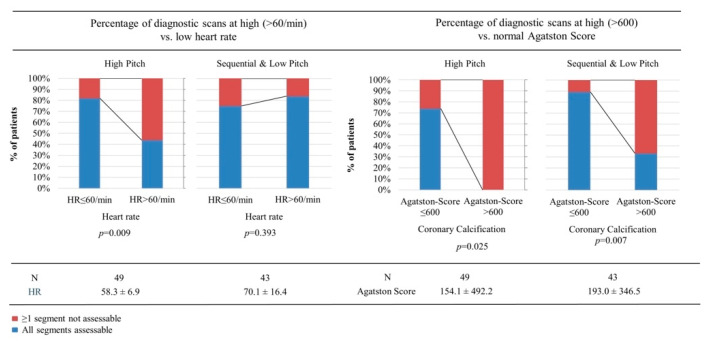
Based on the preceding analysis of diagnostic coronary segments per patient, presented in Figure 3 and Figure 4, an Agatston score of 600 and a heart rate (HR) of 60/min were chosen as cutoffs to compare the percentage of completely diagnostic CCTA scans.

Based on the preceding segment analysis, the selected cutoffs for per-patient analysis were 600 for Agatston score and 60/min for HR. The frequency of non-diagnostic PCD-CCTA scans was higher for Agatston score > 600 using high-pitch (26% vs. 100% non-diagnostic scans, *p* = 0.025), and even sequential and low-pitch modes (11% vs. 67% non-diagnostic scans, *p* = 0.007). A HR > 60/min resulted in a higher frequency of non-diagnostic scans for high-pitch mode (18% vs. 56% non-diagnostic scans, *p* = 0.009), but not for sequential and low-pitch modes (27% vs. 16% non-diagnostic scans, *p* = 0.4) (Figure 5).

At high-pitch mode, the percentage of diagnostic scans was significantly lower at a HR > 60 or an Agatston score > 600 (just three patients with an Agatston score > 600 were scanned with high-pitch mode, all had non-diagnostic scans). At sequential or low-pitch modes, a HR > 60 did not significantly increase non-diagnostic scans, but an Agatston score > 600 was clearly associated with significantly more non-diagnostic scans.

**Figure 6:**
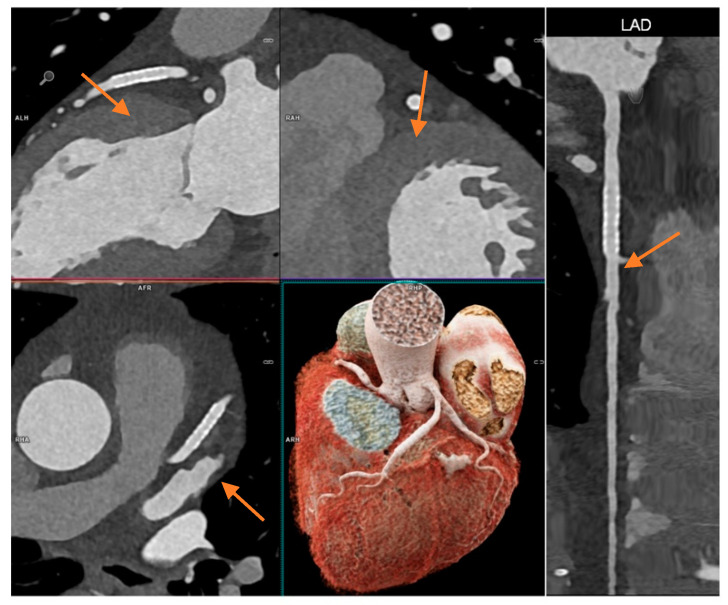
CCTA scan of a 71-year-old female patient with known CAD and prior stenting of the left anterior descending artery (LAD, stent marked with orange arrow). She had unspecific chest discomfort and CCTA was intended to rule out in-stent restenosis. A high-pitch scan was performed at HR 59/min. All segments were diagnostic and had an IQ of one or two. Stent lumen was clearly visible and in-stent restenosis could be excluded with high certainty (2.5 mm stent diameter). Effective radiation dose was 0.76 mSv.

Coronary stents in all patients (*n* = 4) were evaluable. Figure 6 shows in-stent restenosis exclusion by PCD-CCTA.

### 3.7. Diagnostic Performance in the Subgroup with ICA as Reference Standard

Nine patients underwent diagnostic ICA, comprising 126 coronary segments for comparison with ICA as a reference standard (Table 5).

Diagnostic performance for significant CAD was very high for coronary segments (sensitivity 92%, specificity 96%, accuracy 95%). Among the nine patients, there were no false-negative diagnoses per patient but two false-positive diagnoses (accuracy 78%). One patient had coronary stents (*n* = 3) in which significant in-stent-restenosis was correctly excluded.

## 4. Discussion

This study systematically evaluated PCD-CCTA regarding the assessability of coronary arteries and stenosis grading, especially considering calcified plaques, in the context of acquisition protocol, patient size, and heart rate in a retrospective clinical sample. Generally, the image quality was high, leading to a good assessability of coronary segments and vessels even in cases of calcified plaques and stents. The assessability of coronary segments was reduced by a very high calcium burden (Agatston score > 600) and heart rate in high-pitch protocol, as expected. Given the high spatial resolution and contrast-to-noise ratio, accompanied by dose efficiency, PCD-CCTA provides an excellent tool for the evaluation of CAD.

### 4.1. Diagnostic Performance in Clinical Practice

The assessability in the relevant proximal and mid coronary segments >2 mm lumen diameter was excellent (97–100%). As expected, due to physiologic tapering of the coronary arteries, distal segments were more difficult to assess [16]. Considering the feasibility approach of this study, we classified a study as non-diagnostic as soon as one segment was non-diagnostic. Depending on the diameter of this vessel, this may possibly have no effect on therapeutic treatment. Physiologic tapering of peripheral coronary artery segments <2 mm occurs frequently, potentially over-estimating anatomic stenoses that are clinically not relevant for a decision on interventional revascularization [13].

Our collective of patients had a relatively high number of stenoses and calcium burdens. Until today’s technical progress, calcified plaques have relevantly limited image quality due to two different artefacts: blooming artefacts and beam-hardening artefacts [17]. The first-mentioned blooming artefacts are caused by limited spatial resolution and partial volume averaging of different densities within a single voxel. High-density calcification contaminates the density of other tissues in the same voxel as well as in adjacent voxels, and therefore increases the real size of the calcified plaque [18]. Hence, in the past, the overestimation of severely calcified plaques resulted in false-positive diagnoses of CCTA in clinical settings, with a reduction in specificity [19,20,21]. In our study, relevant segments of patients with Agatston scores < 600 remained assessable. This effect is most likely due to the high spatial and temporal resolution, reducing artefacts. The extremely high CNR in spectral imaging compared with other scanners helped to maintain assessability in difficult cases.

In our study, patients’ preparation with beta-blockers resulted in a mean HR of 64 bpm to allow for high diagnostic image quality in a clinical setting. Due to this, image quality dependence on HR may not be representative, as only a few patients with tachyarrhythmia were examined. Thus, this study is not powered for a thorough analysis of the effect of high heart rates using sequential or low-pitch retrospectively gated spiral mode. For the few cases in our study with high heart rates, sequential and retrospective modes resulted in good image quality. Diagnostic accessibility was most relevantly reduced in patients with high-pitch protocols in heart rates > 60 bpm, which may also be a result of the unexpected reflex rise of heart rate after the application of nitrates and high-flow pressure infusion of contrast agent.

### 4.2. Radiation Dose

The radiation dose was generally low. As expected, the radiation dose depended strongly on the scan mode, with a mean effective dose of around 1 mSv for the high-pitch protocol. The good image quality in patients with higher heart rates examined with the sequential mode and retrospective gating was at the expense of significantly higher radiation doses, as expected. However, good image quality should not be sacrificed by inadequately choosing the high-pitch protocol for the sake of dose reduction in patients not suitable for further reduction of heart rate in the context of tachycardia. Compared with 120 kV scans at a third-generation dual-source CT scanner, CCTA radiation dose was similar in our study [12,22,23]. Generally, all other dose-reduction strategies (e.g., automatic tube current modulation and iterative reconstruction) have been performed [24,25].

### 4.3. Diagnostic Performance with ICA as Reference Standard

In comparison with ICA, a small subgroup could outline an accuracy of over 95% of PCD-CCTA on a per-segment level. CT technology advances with improved temporal and spatial resolutions have continuously increased the accuracy and robustness of CCTA for the non-invasive assessment of CAD in the past [26,27]. Several studies demonstrated the ability of CCTA to exclude coronary artery stenosis [28,29] and assess patient outcomes [30]. Nevertheless, the limitations of CCTA, namely in the case of severe coronary artery calcifications, were known, and had to be addressed for further development of CCTA [31,32,33]. Zhang et al. outlined that most errors in the overestimation of calcified coronary artery plaques occurred in large plaques, resulting in a correct prediction of obstructive coronary artery stenosis in only two-thirds in comparison with ICA. In contrast, the prediction of obstructive coronary artery stenosis in small and moderate calcified plaques was accurate in 90% of the cases [34]. A distinctive feature of our study is the high number of significantly calcified coronary segments. Despite this fact, which in the past was considered a clear limitation, our study showed a preserved assessability of coronary artery stenosis up to an Agatston score of 600 and excellent diagnostic performance in the high-risk subgroup with ICA. Patients charged for ICA in our study comprised a highly selected cohort for comparison with CCTA having a higher pre-test probability of significant CAD stenosis, which might render the correct exclusion of relevant CAD more difficult than in the standard collective with intermediate pre-test probability [1]. Despite that, the negative predictive value per segment was 99%, and there were no false-negative diagnoses per patient. This might reassure physicians using PCD-CCTA to exclude relevant CAD in a clinical setting, although the subgroup of patients with ICA was too small for a reliable per-patient analysis. On the other hand, the small number of invasive diagnostic examinations was partly related to the high confidence in non-invasive diagnostic imaging, including PCD-CCTA and functional tests [1].

### 4.4. Limitations

The following study limitations must be acknowledged. Not all advantages of a spectral dataset, e.g., postprocessing with spectral subtraction of coronary calcifications (“pure lumen”), were available at the start of this study. Moreover, ultrahigh-resolution PCD-CCTA with a slice thickness of 0.2 mm is a recent additional opportunity [35]. These options might further enhance the diagnostic assessability and accuracy of calcified plaques. Only a very-limited number of patients had ICA following CCTA, so the comparison of the stenosis degree, and hence the accuracy with the reference standard, was mainly not possible on a per-patient level. Additionally, the study population was relatively young (mean age 58.4 years), which could lead to a possible easily obtainable low HR and better image quality. Further studies should investigate a more heterogenous population to further outline the feasibility of PCD-CCTA in more challenging populations. This study was planned as a feasibility study of the newly established PCD-CCTA, not evaluating hemodynamic relevance and clinical effectiveness considering the outcome. Nevertheless, this multicenter study lays the foundation for further studies analyzing the diagnostic performance of PCD-CCTA.

## 5. Conclusions

High temporal resolution photon-counting detector coronary CTA provides high image quality and CNR with 95% assessability of coronary segments. The assessability was limited by very excessive coronary calcifications or high heart rates in high-pitch mode. For a subgroup with ICA, the diagnostic accuracy per segment was very high, although the limited number of patients who underwent both diagnostic modalities limits the generalization of this particular finding at this stage.

## Figures and Tables

**Table 1:** Baseline characteristics of clinical and scan parameters are given.

	Mean ± SD or N (%)
Age (years)	58.4 ± 13.7
Male gender	60 (65%)
History of CAD	8 (8.9%)
History of stenting	4 (4.3%)
History of coronary artery bypass graft (CABG)	0
History of myocardial infarction (MI)	0
Hyperlipidemia	38%
Hypertension	59%
Smoking	17%
Diabetes mellitus	14%
BMI (kg/m²)	26.9 ± 5.9
Agatston score	172.3 ± 428.5
kV average (kV)	120 ± 0
Effective mAs (mAs)	52.5 ± 29.6
CTDI.vol (mGy)	16.4 ± 25.3
Dose length product (mGy.cm)	234.1 ± 347.6
Effective dose (mSv)	3.51 ± 5.21
Contrast volume (mL)	70.9 ± 8.3
Contrast flow (mL/s)	5.3 ± 0.5
Received beta-blockers	72 (78%)
Average heart rate (bpm)	63.8 ± 13.6
Minimum heart rate (bpm)	62.8 ± 29.3
Maximum heart rate (bpm)	68.1 ± 19.3
HR variability	0.095 ± 0.285
Right coronary artery dominance	61 (66%)
Co-dominance of coronary arteries	18 (20%)
Left coronary artery dominance	13 (14%)

**Table 2:** Total assessability (considering any reason rendering segments non-diagnostic, such as movement artifacts or extensive calcifications), as well as median and mean image quality of all coronary segments, are listed.

Coronary Artery	Segments	Assessability (%)	Image Quality (Mean ± SD)	Image Quality (Median, (IQR))
Overall	all	95	1.70 ± 0.76	1 (IQR 1–2)
RCA	1	98	1.37	1
	2	96	1.62	1
	3	97	1.69	1
	4	88	1.90	1
LAD	5	100	1.18	1
	6	96	1.21	1
	7	98	1.49	1
	8	97	1.95	2
	9	94	2.05	2
	10	95	1.84	1
LCX	11	97	1.45	1
	12	93	1.87	1
	13	93	1.85	1
	14	92	2.00	1
	15	87	2.33	2
***p-value***		<0.001	<0.001	<0.001

RCA (right coronary artery), LAD (left anterior descending artery), LCX (left circumflex artery).

**Table 3:** Image quality and assessability of coronary vessels are given, considering the overall frequency of diagnostic coronary vessels regarding any reason of image degradation (% Diagnostic), frequency of diagnostic coronary vessels considering only calcified coronary plaques (% Diagnostic CAC), and frequency of diagnostic coronary vessels considering only movement artefacts and low contrast (% Diagnostic Mov.).

Coronary Artery	IQ (Median, IQR)	CNR (Mean ± SD)	Agatston-Score (Mean ± SD)	Diagnostic (%)	Diagnostic CAC (%)	Diagnostic Mov. (%)
LAD	1 (1–2)	20.65 ± 10.01	79.84 ± 210.34	87	92	95
LCX	1 (1–3)	19.86 ± 10.09	24.37 ± 86.50	87	95	90
RCA	1 (1–2)	20.44 ± 11.35	59.43 ± 169.73	86	93	90
***p-value***	0.007	0.041	0.001	0.951	0.549	0.431

IQ (image quality), CNR (contrast-to-noise-ratio), CAC (coronary artery calcium), Mov. (movement).

**Table 4:** Clinical parameters are compared between patients with and without totally diagnostic CCTA scans to examine a potential influence on total CCTA scan assessability.

	Assessability at High-Pitch			Assessability Sequential and Low-Pitch		
	All Segments Assessable (*n* = 34; 69%)	≥1 Segment Not Assessable (*n* = 15; 31%)	*p*-Value	All Segments Assessable (*n* = 35; 81%)	≥1 Segment Not Assessable (*n* = 8; 19%)	*p*-Value
BMI [kg/m^2^] (mean ± SD	25.3 ± 2.4	26.7 ± 8.2	0.402	26.2 ± 5.7	33.6 ± 7.4	0.013
HR [bpm] (mean ± SD	57.2 ± 6.8	60.9 ± 6.6	0.044	70.8 ± 15.9	66.8 ± 19.5	0.267
HV (mean ± SD)	0.035 ± 0.052	0.059 ± 0.116	0.164	0.118 ± 0.401	0.314 ± 0.393	0.110
Agatston Score (mean ± SD)	48.2 ± 107.7	392.9 ± 845.7	0.011	125.9 ± 265.7	486.6 ± 506.4	0.043
Age (mean ± SD)	56.1 ± 14.2	56.7 ± 15.4	0.446	60.9 ± 12.5	60.0 ± 14.1	0.430
Male Gender (%)	68%	76%	0.474	50%	57%	0.507

BMI (body mass index), HR (heart rate), HV (heart rate variability).

**Table 5:** Nine patients (high-pitch *n* = 2; sequential *n* = 6; low-pitch *n* = 1) underwent invasive coronary angiography (ICA) after CCTA. These included 126 coronary segments. Per-segment and per-patient diagnostic performance parameters are shown.

**Per Segment**	**For Coronary Stenosis > 50%**
Total Number (*n*)	**126**
Significant Stenosis (≥50%)	13
No Significant Stenosis	113
Segments with Stents	3 (all evaluable)
True Negative	108
True Positive	12
False Negative	1
False Positive	5
Sensitivity	92%
Specificity	96%
PPV	71%
NPV	99%
Accuracy	95%
**per patient**	**For coronary stenosis >50%**
Total Number (*n*)	**9**
Significant Stenosis (≥50%)	3
No Significant Stenosis	6
Patients with Stents	1 (evaluable)
True Negative	4
True Positive	3
False Negative	0
False Positive	2
Sensitivity	100%
Specificity	67%
PPV	60%
NPV	100%
Accuracy	78%

PPV (positive predictive value), NPV (negative predictive value).

## Data Availability

The data presented in this study are available on request from the corresponding author.

## Supplementary Materials

The following supporting information can be downloaded at: https://www.mdpi.com/article/10.3390/jcm11206003/s1, Table S1: Multiple regression analysis with the image quality of whole PCD-CCTA scans as the outcome of interest and gender, age, BMI, HR, HV and Agatston- score, as covariates yielded only HR and HV as significant predictors of IQ in high-pitch mode but not in sequential mode.
