# Supplementary material for: High Temporal Resolution Dual-Source Photon-Counting CT for Coronary Artery Disease: Initial Multicenter Clinical Experience
Source: J Clin Med. 2022 Oct 11;11(20):6003. doi: 10.3390/jcm11206003 (PMC9604695; doi:10.3390/jcm11206003)
Supplement: Supplementary file 1 [file jcm-11-06003-s001.zip › jcm-1926526-supplementary.pdf]

Table S1a. High Pitch Mode

	Image Quality	
Linear Regression	***β***	***p-value***
Male Gender	-0.033	0.862
Age (per 10 years)	-0.432	0.055
BMI	-0.048	0.794
Heart Rate	0.378	0.074
HR Variability	0.613	0.007
Agatston-Score	0.211	0.298

Multiple linear regression; p-value model = 0.012.

Table S1b. Sequential and Low Pitch Mode

	Image Quality	
Linear Regression	β	p-Value
Male Gender	0.225	0.195
Age (per 10 years)	-0.107	0.541
BMI	0.316	0.092
Heart Rate	0.220	0.214
HR Variability	-0.123	0.459
Agatston-Score	0.226	0.237

Multiple linear regression, p-value model = 0.149.
